# Influence of geographical environment on retinol-binding protein reference values in Chinese men

**DOI:** 10.1371/journal.pone.0297204

**Published:** 2024-01-24

**Authors:** Peng Wei, Miao Ge, Lei Zhang

**Affiliations:** Institute of Health Geography, School of Geography and Tourism, Shaanxi Normal University, Xi’an, China; University 20 Aout 1955 skikda, Algeria, ALGERIA

## Abstract

This study combined geographic factors to predict Chinese healthy male RBP reference values from a geographic perspective, with the aim of exploring the spatial distribution and regional differences in Chinese healthy male Retinol-Binding Protein(RBP) reference values, and then providing a theoretical basis for medical diagnosis of healthy male RBP reference values in different regions of China. Using the actual measured RBP values of 24,502 healthy men in 256 cities in China combined with 16 geographical factors as the base data, the spatial autocorrelation, correlation analysis and support vector machine were used to predict the RBP reference values of healthy men in 2322 cities in China, and to generate a spatial distribution map of the RBP reference values of healthy men in China. It was found that the spatial distribution of healthy male RBP reference values in China showed a trend of gradual increase from the first to the third terrain steps. Combined with the distribution map, it is suggested that the RBP reference values of healthy men in China should be divided into the low value zone of the first-level terrain step (25mg/L~40mg/L), the middle value zone of the second-level terrain step (40mg/L~45mg/L) and the high value zone of the third-level terrain step (45mg/L~52mg/L).

## Introduction

Retinol-binding protein(RBP) is a small molecule protein in plasma, a specific transporter protein of vitamin A in blood, specifically synthesized by the liver, metabolized mainly in the kidney, and widely distributed in blood, cerebrospinal fluid, urine and other body fluids [1, 2]. The level of the reference value of RBP is important for the detection of early functional impairment of the renal tubules and can sensitively reflect the degree of damage to the renal proximal tubules [3]. Therefore, RBP is often used as an important indicator to monitor the degree of early renal impairment, and also as an indicator of early hepatic impairment and monitoring therapy [4]. An elevated RBP reference value indicates that the body is at risk of renal insufficiency or overnutrition of fatty liver [5]. Decreased RBP reference values indicate the risk of vitamin A deficiency, hypoproteinemia, liver disease and malabsorption syndrome [6–8]. Previous studies have focused on the effects of age, gender and ethnicity on RBP values. Studies [9, 10] found that the in vivo levels of RBP was correlated with age and gender, being lower in women than in men and lower in children than in adults, but there were no gender differences in children. Yilihamu Abulitif found no significant differences in RBP reference values between different ethnic groups in China [11]. As China is a vast country with diverse climate types and environmental changes; therefore, the range of diagnostic marker RBP reference values needs to be tailored to local conditions for better monitoring, diagnosis and prevention of chronic diseases. However, studies have been mainly limited to a small region and differences in RBP reference values in different regions are not fully developed [12]. Therefore, this study used different analytical methods combined with spatial statistical techniques to demonstrate the spatial distribution pattern and regional differences of RBP reference values in healthy men in China, to improve the environmental evaluation system of RBP reference values in different regions of China, and to reclassify them into intervals, which will in turn provide a scientific basis for the prevention of chronic diseases, such as impaired liver [13], renal tubular function abnormalities [14], hyperthyroidism [15], and hypoproteinemia diseases [16], in China.

## Materials and methods

### Data source

The statistical data applied in this study mainly include includes two parts, which are medical statistical data and geographical data. The medical statistics refer to the medical reference values of RBP of 24,502 healthy men from 256 cities in China measured by China Knowledge Network, Wanfang database, China Science Citation Database, Super Star Library and some hospitals(See data set at the end of the article); the geographic statistics are the data of 16 geographic factors from the shared information of China National Mapping Data Center(https://www.ngcc.cn/ngcc/html/1/391/392/16114.html), China Meteorological Data Sharing Service Network(http://data.cma.cn) and World Harmony Soil Database(Harmonized World Soil Database, HWSD; http://www.fao.org/nr/land/soils/harmonized-world-soil-database/zh/), respectively. Currently, national and international studies have shown that geographic location [17], temperature [18] and soil [19] affect RBP reference values. Therefore, in this study, 16 geographic factors were specifically chosen to explore their relationship with RBP reference values, and the 16 geographic factors are as follows: X_1_ longitude (°), X_2_ latitude (°), X_3_ altitude (m), X_4_ annual sunshine hours (h), X_5_ annual average temperature (°C), X_6_ annual temperature difference (°C), X_7_ annual precipitation (mm), X_8_ annual average relative humidity (%), X_9_ annual average wind speed (m/s), X_10_ topsoil clay percentage (%wt), X_11_ topsoil silt percentage (%wt), X_12_ topsoil reference capacity (g/cm3), X_13_ topsoil organic matter content (%wt), X_14_ topsoil pH, X_15_ topsoil (clay) cation exchange capacity (cmol/kg), X_16_ topsoil (silt) cation exchange capacity (cmol/kg).

### Analyze ideas

**Fig 1:**
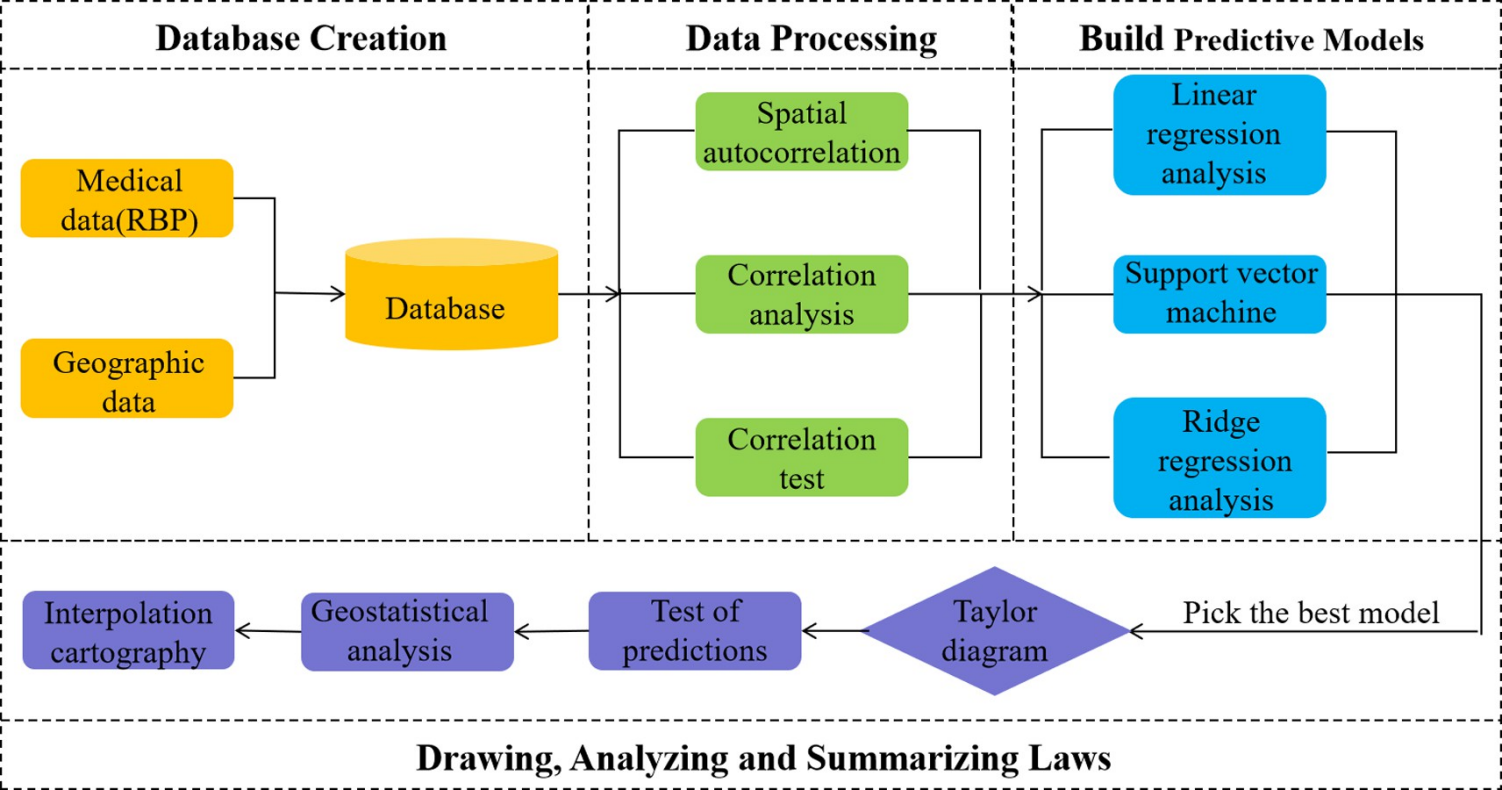
Flow chart of the study.

The flow chart of this study is as follows (Fig 1). First, the medical data and geographic environment data are matched to create a database. Then different data analysis methods were used to filter out the appropriate geoenvironmental factors. Next, three models were constructed to predict the nationwide RBP reference values. Finally, the spatial distribution of RBP reference values in China was plotted and the prediction results were combined to make a secondary division of the RBP reference value interval in China.

## Results

### Spatial autocorrelation

**Fig 2:**
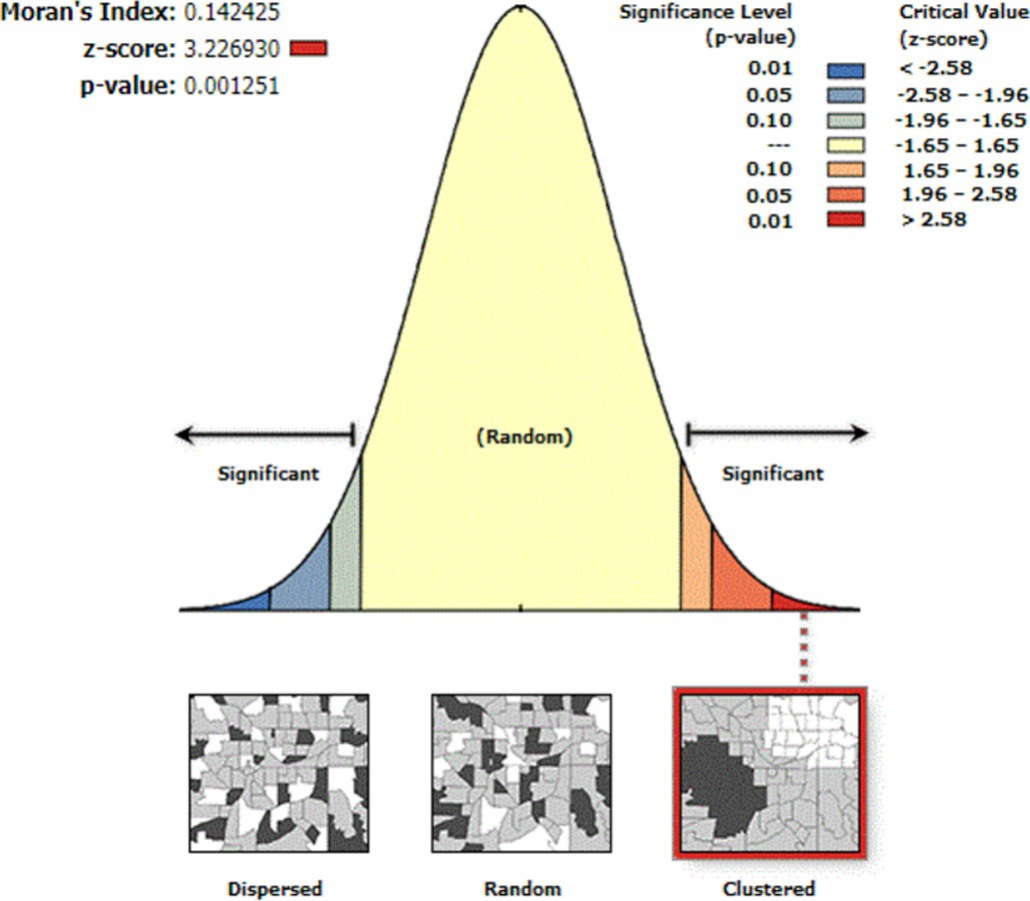
Spatial autocorrelation analysis of the RBP sample data.

The theory of spatial autocorrelation was proposed by Professor Kindu Tobler based on the first law of geography, specifically by going to reveal the distribution of spatial data and then calculating the degree of correlation of things in spatial autocorrelation to indicate the potential interconnection between things [20]. According to the scope of the spatial units studied, spatial autocorrelation can be specifically divided into global spatial autocorrelation and local spatial autocorrelation, and the difference between them mainly lies in the continuity and wholeness of the spatial units studied. Since this paper is about the reference value of RBP in China, the global spatial autocorrelation is chosen for this study, which includes three evaluation indexes, Moran’s *I*, *Z* score and *P* value. Combined with the spatial autocorrelation results (Fig 2), it can be found that the Moran’s *I* index of RBP sample data is 0.142, which is between [–1,1] and greater than 0. This means that the distribution of RBP sample data in China has the same trend as the distribution of certain geographical factors in China; the global autocorrelation coefficient *Z* is 3.227, which is greater than its |*Z*| = 2.54 in the 0.01 confidence interval, indicating that the spatial autocorrelation of RBP sample data in our spatial region is significant; the significance *P* = 0.001<0.01, indicating that there is an extremely significant relationship between RBP sample data and geographic factors in various places. Combining Moran’s *I* index, *Z* value and *P* value, it was found that the distribution of RBP reference values in China had strong spatial autocorrelation and showed high value clustering, so that follow-up studies could be conducted.

### Correlation analysis and test

Correlation analysis was proposed by the famous British statistician Francis Galton in the seventeenth century to specifically study the relationship between variables and is mainly applied in data dimensionality reduction, machine learning data preprocessing and outlier determination analysis [21]. The RBP reference values were found to be correlated with 7 geographical factors(Table 1), with X_7_ annual average precipitation and X_12_ topsoil reference capacity in general, and X_1_ longitude, X_3_ altitude, X_9_ annual average wind speed, X_10_ topsoil clay percentage and X_15_ topsoil (clay) cation exchange insignificant correlation. There is a significant correlation between the cation exchange capacity. The order of the correlation coefficients between the seven geographic factors and the RBP reference value is as follows: X_1_ longitude>X_3_ altitude>X_9_ annual average wind speed>X_15_ topsoil (clay) cation exchange capacity>X_10_ topsoil clay percentage>X_7_ annual average precipitation> X_12_ topsoil reference capacity. It can also be found that the correlation coefficients of X_1_ longitude, X_7_ annual average precipitation, X_9_ annual average wind speed, X_12_ topsoil reference capacity and X_15_ topsoil (clay) cation exchange capacity are greater than 0, that is, there is a positive correlation between these five geographical factors and the RBP reference value; The correlation coefficient between X_3_ altitude and X_10_ topsoil clay percentage is less than 0, that is, there is a negative correlation between these two geographic factors and the RBP reference value.

**Table 1:** Correlation analysis between geographical factors.

Geographical factors	*r*	*P*	Geographical factors	*r*	*P*
**X**_**1**_	0.576**	0.000	X_9_	0.497**	0.000
**X**_**2**_	-0.072	0.380	X_10_	-0.267**	0.001
**X**_**3**_	-0.520**	0.000	X_11_	0.048	0.564
**X**_**4**_	0.041	0.617	X_12_	0.191*	0.019
**X**_**5**_	0.148	0.070	X_13_	-0.125	0.128
**X**_**6**_	-0.003	0.973	X_14_	0.116	0.157
**X**_**7**_	0.240*	0.003	X_15_	0.273**	0.001
**X**_**8**_	0.152	0.064	X_16_	-0.018	0.829

* represents correlation

** represents significant

**Fig 3:**
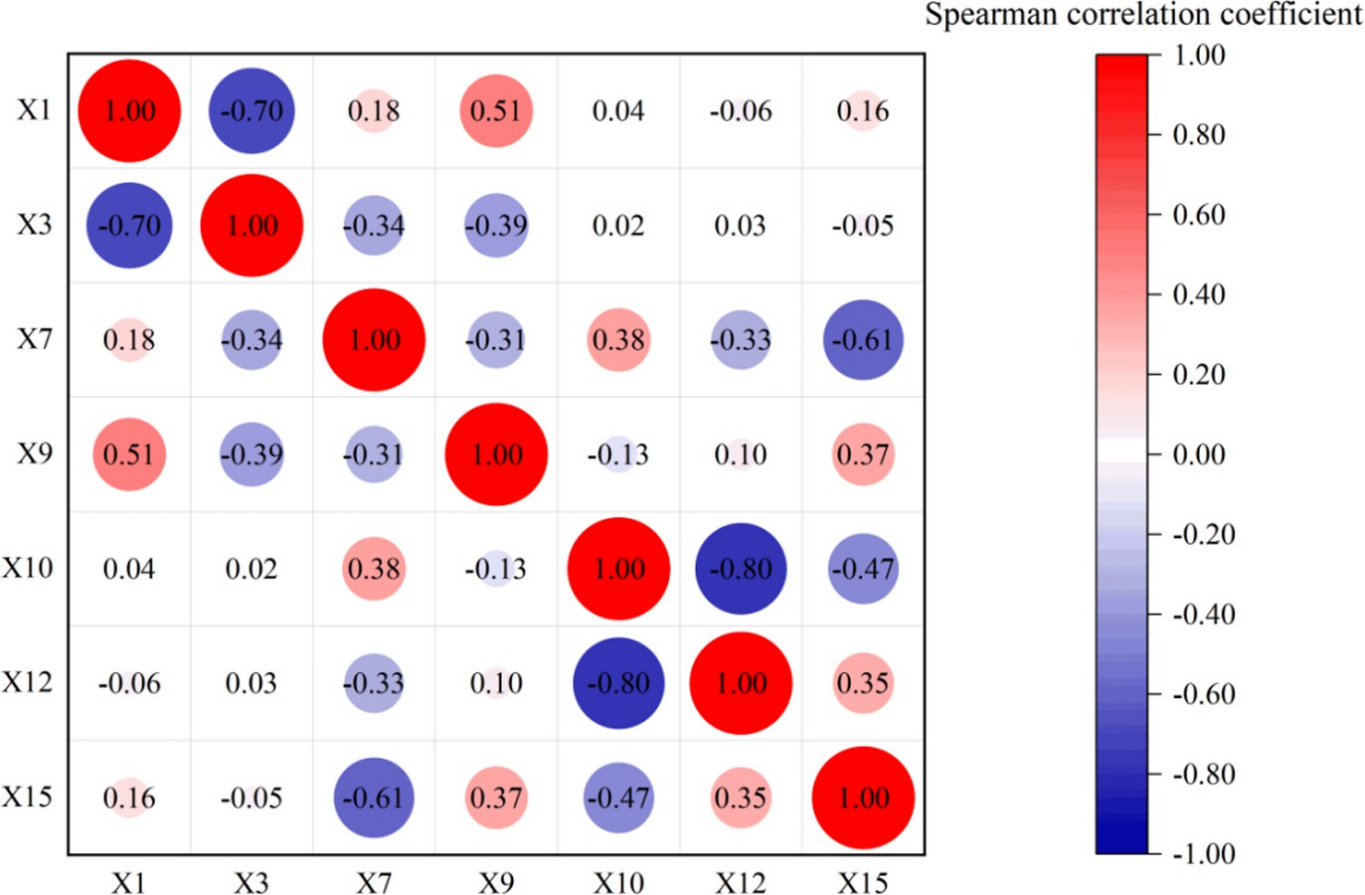
Correlation test for geographical factors.

The Spearman correlation coefficients between these seven geographic factors correlated with the RBP reference values were used to depict the heat map (Fig 3), with red representing positive correlations and blue representing negative correlations, and the darker the color in the same color indicates that the variables are more correlated with each other, and the higher the correlation, the stronger the covariance between the variables, and the lighter the color, the opposite. Meanwhile, the size of the circle area in the graph also indicates the correlation degree between the factors, the larger the circle area, the stronger the correlation degree, and the smaller the circle area, the weaker the correlation degree. It is generally considered that when the correlation between two variables reaches 0.9 or more, it indicates that there is a strong covariance between these two variables, and then the factors should be traded off to ensure the accuracy of subsequent modeling [22]. It can be found in Fig 2 that the correlation coefficients between these seven geographic factors do not reach 0.9, indicating that there is no strong covariance problem between these seven geographic factors, and all of them can be included in the subsequent study for modeling prediction.

### Linear regression analysis

Linear regression analysis is a statistical method used to study the linear relationship between the dependent variable and multiple independent variables [23]. Specifically, SPSS software was used and the reference value of RBP for healthy Chinese men was entered with seven geographical factors, and after linear regression it could be found that none of the seven geographical factors had been removed and all requested variables had been entered in the results. Thus the statistical equation can be obtained as follows: Y = 0.343X_1_-0.00181X_3_+0.00243X_7_+0.6

50X_9_-0.0415X_10_-2.725X_12_+0.0410X_15_±4.874.

### Support vector machine

**Fig 4:**
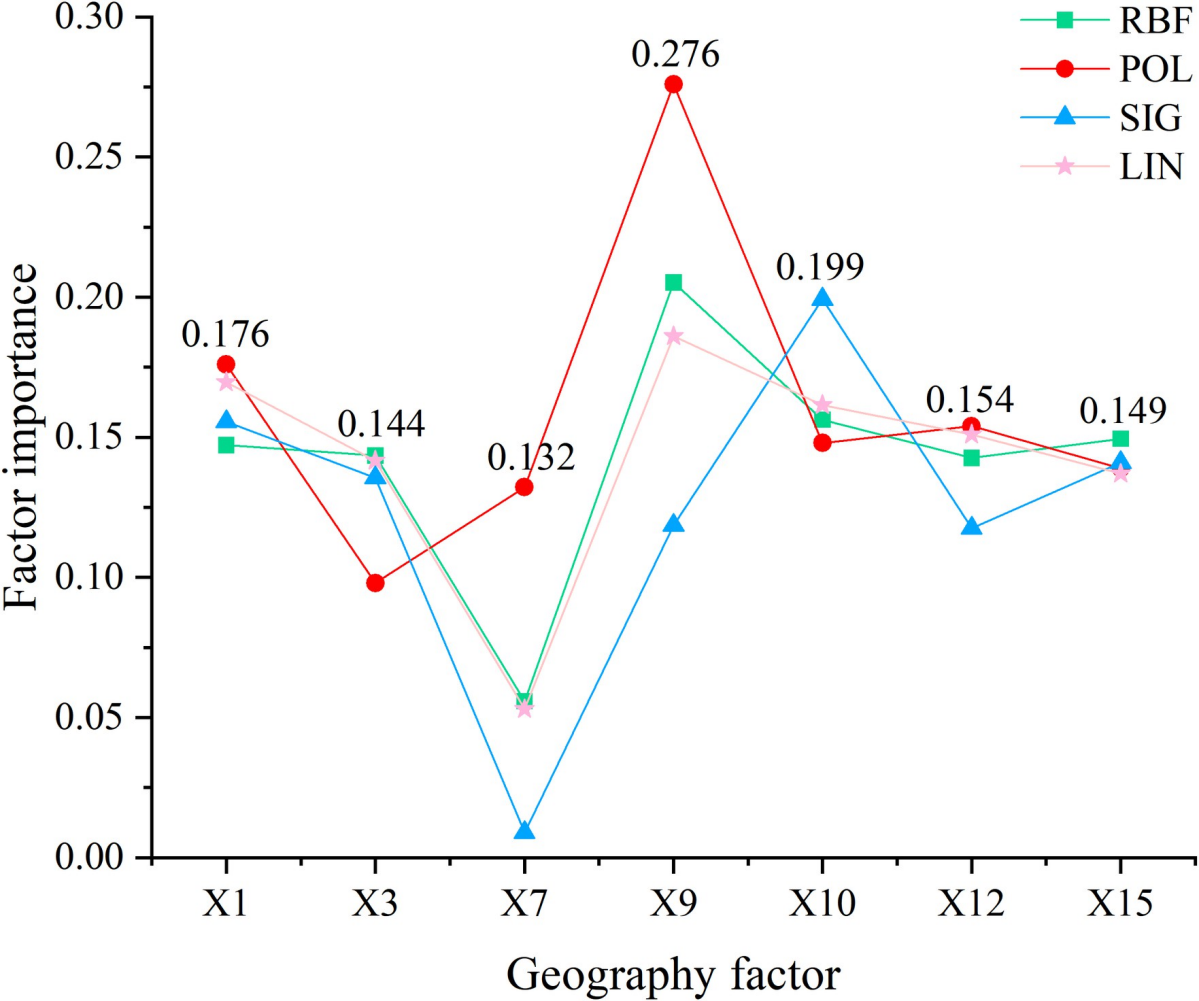
SVM variable importance of RBP.

Support vector machine (SVM) is a binary classification model whose basic principle is structural risk minimization, and SVM has gradually developed into a supervised learning model after combining with mathematical and statistical theories [24]. With the advantages of fast convergence, high generalization ability and global optimality, support vector machines are now widely used in pattern recognition, classification, and regression analysis [25]. In SPSS Clementine 12.0, the RBF kernel function, Polynomial kernel function, SIG kernel function and Linear kernel function are specifically chosen to construct the final prediction model by combining the characteristics of support vector machine models. First, the RBP reference value is selected as the output variable in the Fields setting, and the seven geographic factors associated with the RBP reference value are selected as the input variables, the specific method is selected as the expert method, and then the RBF kernel function, POL kernel function, SIG kernel function and LIN kernel function are selected for SVM training, and the importance diagrams of variables with different kernel functions are selected for output (Fig 4). The horizontal coordinates in the figure represent each geographic factor, and the vertical coordinates represent the variable importance of the factors; the green line represents the RBF kernel function, the red line represents the POL kernel function, the blue line represents the SIG kernel function, and the pink line represents the LIN kernel function. The factor importance of the RBF kernel function on X_3_ and X_15_ is 0.144 and 0.149, respectively; the factor importance of the POL kernel function on X_1_, X_7_, X_9_ and X_12_ is 0.176, 0.132, 0.276 and 0.154, respectively; the factor importance of the SIG kernel function on X_10_ is 0.199; the LIN kernel function performs poorly in terms of each geographical factor importance. Therefore, we finally choose the POL kernel function for SVM prediction modeling of RBP values.

### Ridge regression analysis

**Fig 5:**
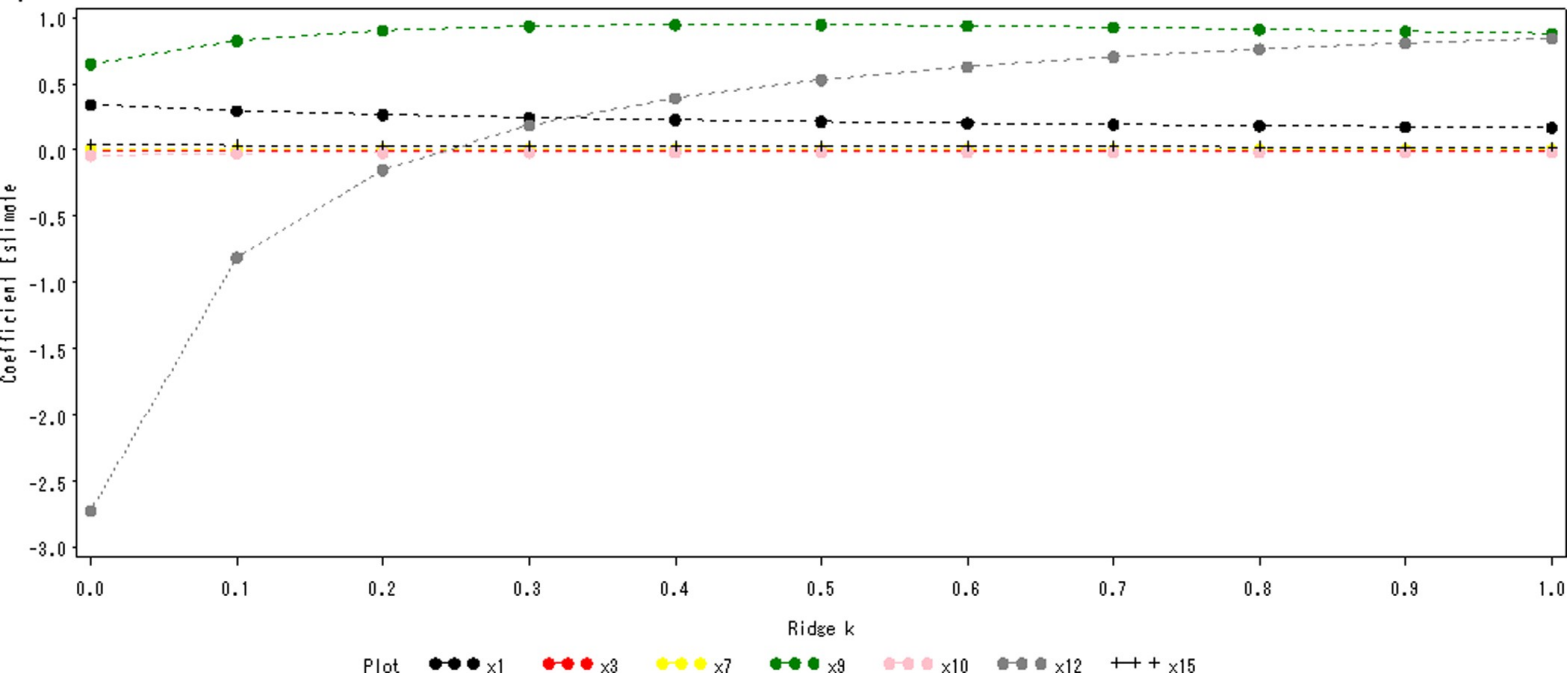
Ridge plot of the RBP.

Ridge regression analysis was introduced by Hoerl and Kennard in 1970 as a regression method by improving the least-squares estimation method [26]. Ridge regression analysis is particularly important to select the appropriate ridge parameter *k* in ridge regression analysis because it is designed to reduce multicollinearity and improve the accuracy of the prediction model by introducing a ridge parameter *k* (0 < *k* < 1), which makes the model prediction more relevant to the actual situation. In SAS, a ridge regression model was constructed by combining the programmed code with the RBP reference value as the dependent variable and the seven geographic factors as the independent variables to obtain a ridge trace (Fig 5). According to the ridge trace diagram, when the ridge parameter *k*≥0.4, the ridge trace of each geographic factor tends to level off gradually, indicating that the prediction error of the ridge regression equation is the smallest and the accuracy is the highest, therefore, *k* = 0.4 is chosen to establish The ridge regression equation of RBP.Y = 13.467+0.229X_1_-0.00264X_3_+0.00184X_7_+0.948X_9_-0.0163X_10_+0.392X_12_+0.0275X_15_±6.662.

### Selection of the optimal model

**Fig 6:**
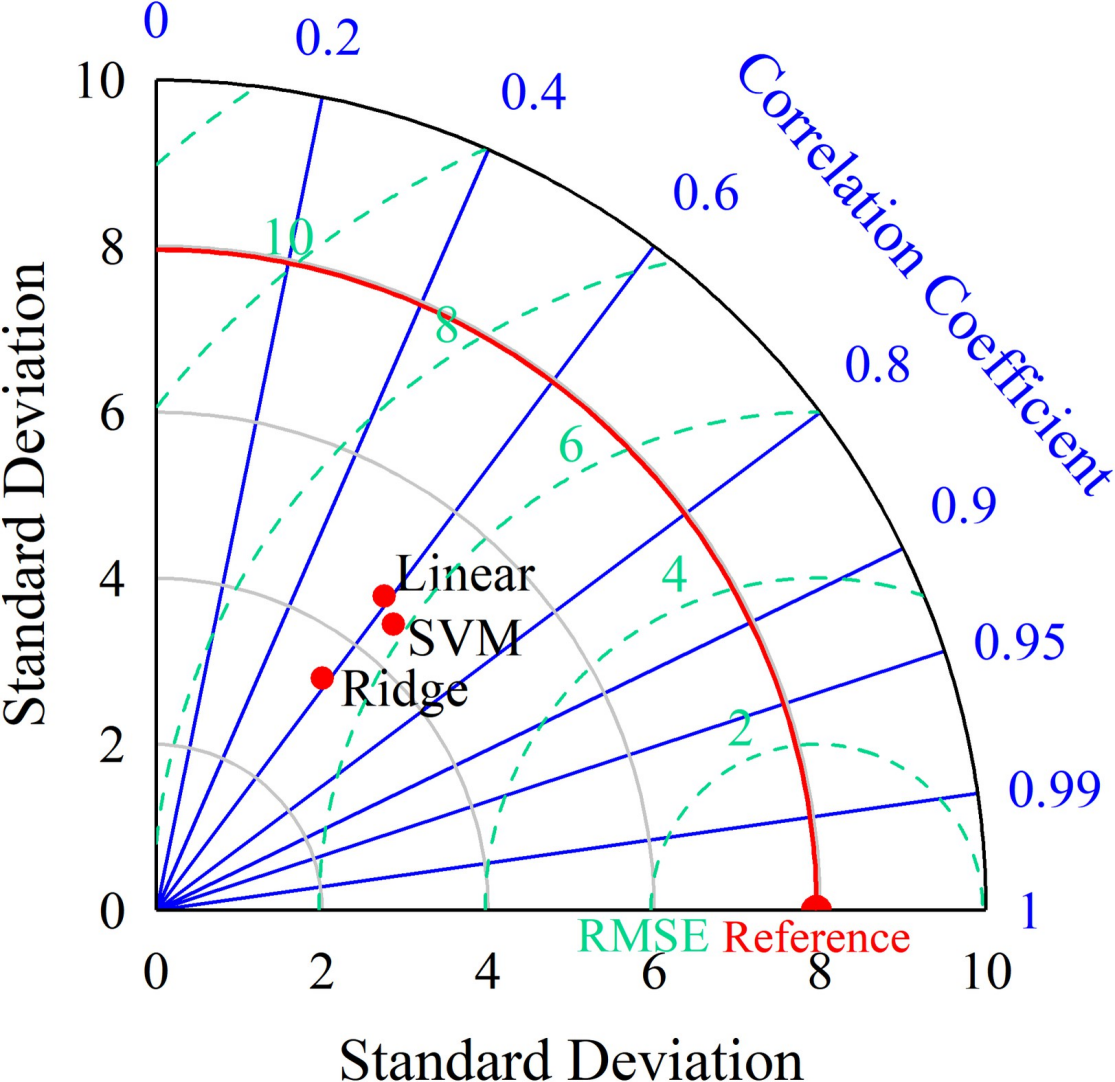
A Taylor plot of the RBP prediction model.

The Taylor diagram was proposed by Karl E. Taylor [27, 28] in 2001 as a method that allows intuitive model comparison. The core idea of Taylor plotting is to design a polar coordinate with only the first quadrant, and cleverly use the geometric principle of trigonometric function to integrate three indicators for evaluating models, correlation coefficient, root-mean-square error and standard deviation, by transforming them into polar and coefficient coordinates on a single plot. The red scatter points represent the individual models, the blue radial lines represent the correlation coefficients, the black horizontal and vertical axes represent the standard deviations, and the green dashed lines represent the root-mean-squared errors. The larger the correlation coefficient, the closer the model prediction is to the measured value, and the higher the model prediction accuracy; the smaller the correlation coefficient, the opposite. The smaller the root-mean-square error, the closer the predicted value of the data is to the measured value through modeling, and the higher the prediction accuracy of the model. The smaller the standard deviation, the closer the predicted value of the model is to the measured value, and the higher the prediction accuracy of the model. represents the standard deviation, the green dashed line represents the root-mean-square error, and the red solid line represents the RBP sample data reference value. The standard deviation of the SVM prediction model is 4.479, the correlation coefficient is 0.638, and the root-mean-square error is 6.138 (Fig 6). A comprehensive comparison shows that the SVM prediction model performs better in all three evaluation indexes, indicating that the SVM prediction model has the highest accuracy in RBP prediction. Therefore, the SVM prediction model was selected to predict the RBP values of 2322 cities in China, and the results of some cities after using the SVM prediction model for prediction were shown (Table 2).

**Table 2:** Prediction value of RBP in some cities.

City	Geographical factors							measured value (mg/L)	SVM predicted value (mg/L)
	longitude (°)	height above sea level (m)	annual mean precipitation (mm)	annual average wind speed (m/s)	topsoil clay particle percentage(%wt)	table soil reference capacity (g/cm^3^)	cation exchange amount of surface clay (cmol/kg)		
**Beijing**	116.07	31.3	571.9	2.5	9	1.6	54	47.62	48.10
**Xi’an**	108.97	397.5	553.3	2.0	21	1.38	34	43.39	45.34
**Urumqi**	87.58	935.0	286.3	2.6	26	1.38	52	33.46	35.58
**Shanghai**	121.39	2.8	1007.3	3.9	6	1.66	109	50.00	49.80
**Chengdu**	104.04	506.1	997.6	1.5	21	1.38	34	42.30	40.33

### Test of prediction results

**Fig 7:**
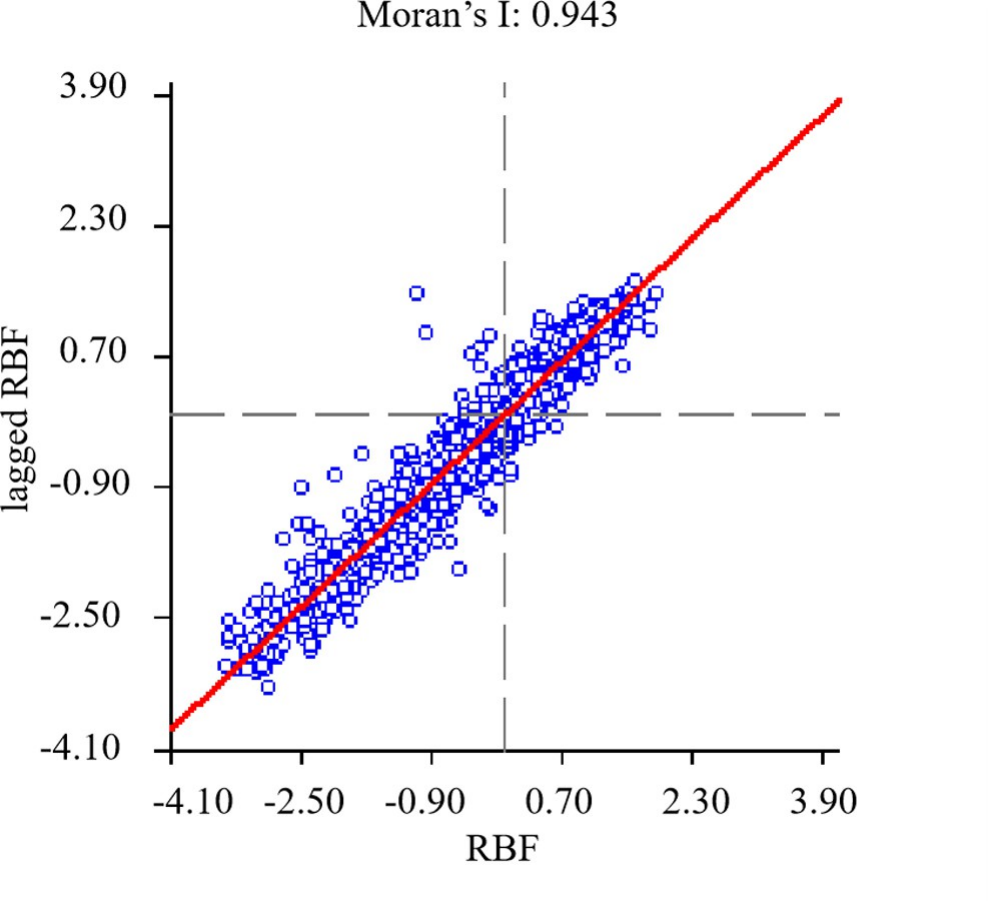
Moran plot of RBP predicted values.

GeoDa spatial correlation and scatter plot were used to test the prediction results. In the GeoDa spatial correlation test, Moran’s index was used to determine the aggregation. a Moran’s I greater than 0 indicates that things are positively correlated in space and tend to be aggregated; a Moran’s I less than 0 indicates that things are negatively correlated in space and tend to be discrete. The male RBP prediction values of 2322 cities nationwide obtained by SVM model prediction were matched with cities in Arcgis, and the shp file was created, and the shp file was imported into GeoDa to calculate the spatial weight matrix, specifically choosing Queen connection, selecting the rank value of adjacency as 1, adding the variables, and then performing global spatial autocorrelation to obtain Moran’s I index and Moran’s scatter plot (Fig 7). The results show that the Moran’s I = 0.943 for the RBP predicted values indicates that the spatial distribution of the RBP predicted values in China is highly clustered, and 0.943 is between [–1,^1^] and closer to 1. Therefore, the RBP predicted values not only have a clustered spatial distribution but also have a strong positive spatial correlation. This corresponds to the high clustering of RBP sample data, indicating that the prediction is more accurate.

**Fig 8:**
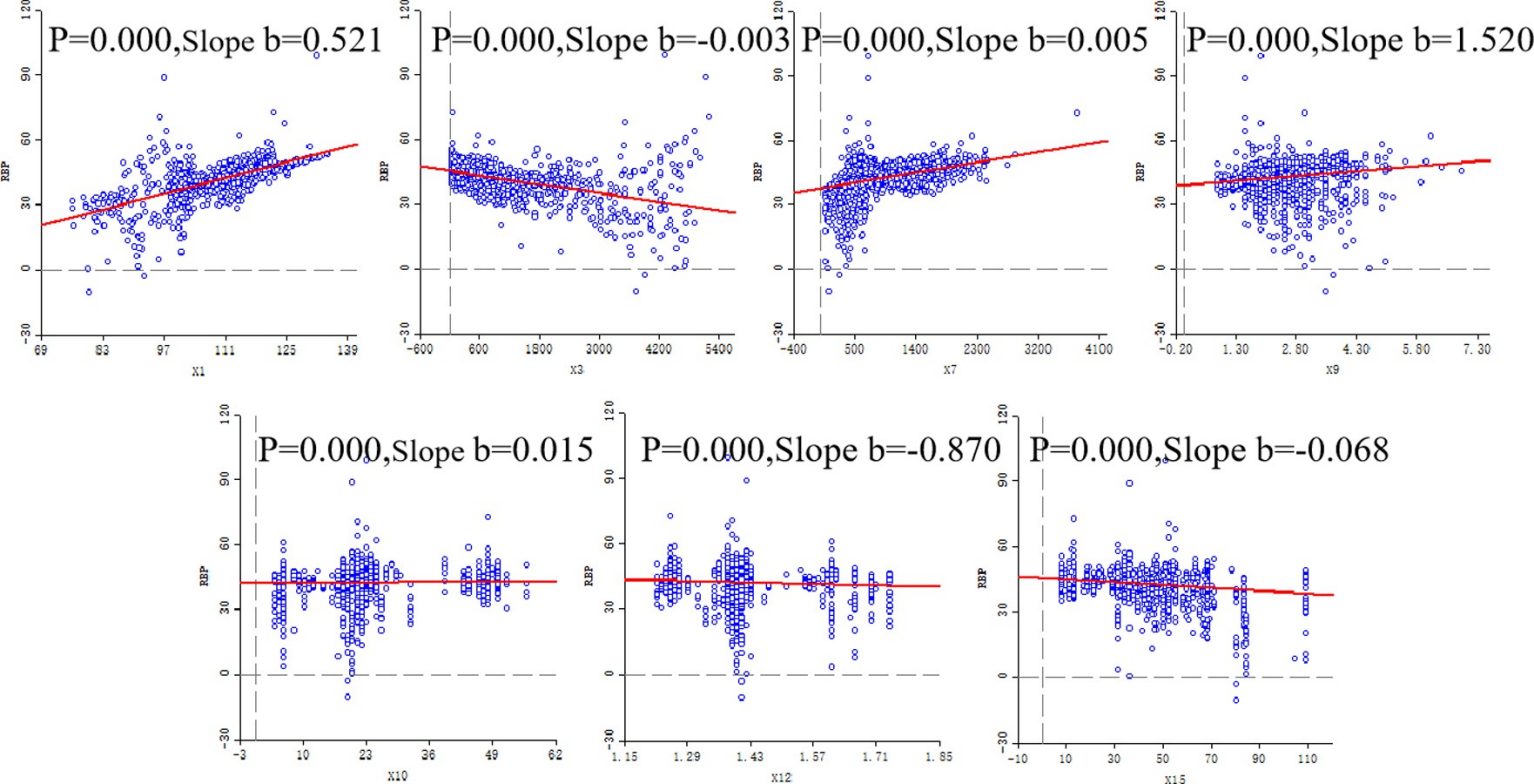
Scatter plot of RBP predicted values versus geographic factors.

In GeoDa, scatter plot variables were explored between the RBP predicted values of 2322 cities and the seven geographic factors selected for modeling, with the RBP predicted values as the dependent variable and X1, X3, X7, X9, X10, X12, and X15 as independent variables to draw scatterplots (Fig 8). Combined with the scatter plots, it can be found that there is a correlation between all these seven geographic factors involved in modeling and the RBP reference values, indicating that the geographic factors involved in modeling are correctly selected.

### Geographical analysis

**Fig 9:**
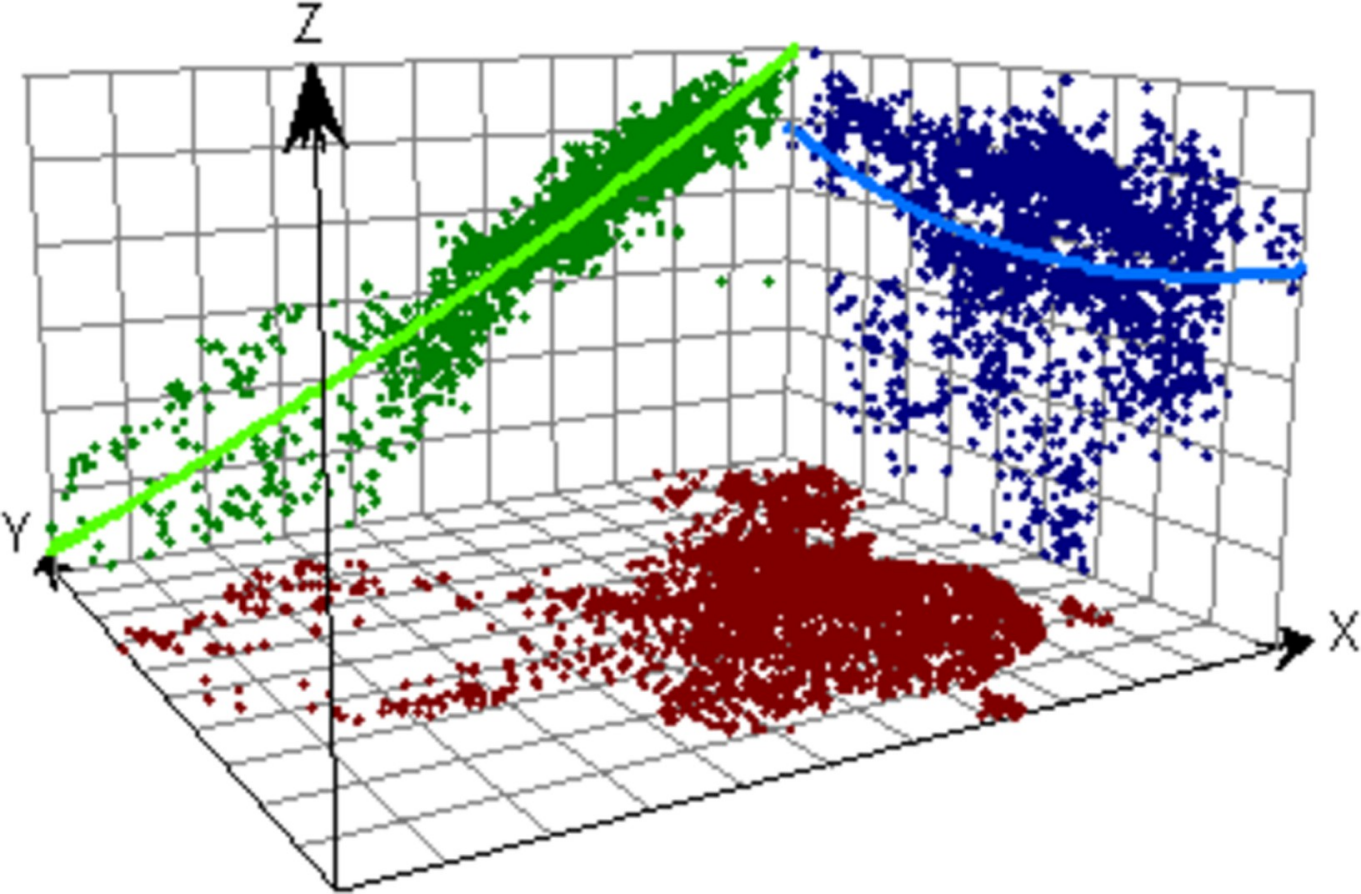
Trend surface analysis of the predicted RBP values.

Geostatistical analysis, which can also be called geostatistics, is a subdiscipline based on many theories proposed by the French statistician Georges Matheron [29], which is specifically based on the theory of regionalized variables, combined with variational functions to study geographic phenomena with certain spatial patterns. The trend analysis diagram is drawn in ArcGIS software. In the trend analysis diagram, the brown points in the diagram are the 2322 urban sample points, and the green points and blue points are the projections of the 2322 urban sample points on the *ZX* plane and *YZ* plane, respectively, and the green curve indicates the projection trend of the healthy male RBP prediction data in the east-west direction in China, and the blue curve indicates the projection trend of the healthy male RBP prediction data in the north-south direction in China. Through the green curve, it can be seen that the RBP prediction values show a sharp increase in distribution from the western region to the eastern region; through the blue curve, it is found that the RBP prediction values first gradually decrease and then gradually increase from north to south, and the overall change trend is not significant (Fig 9).

**Fig 10:**
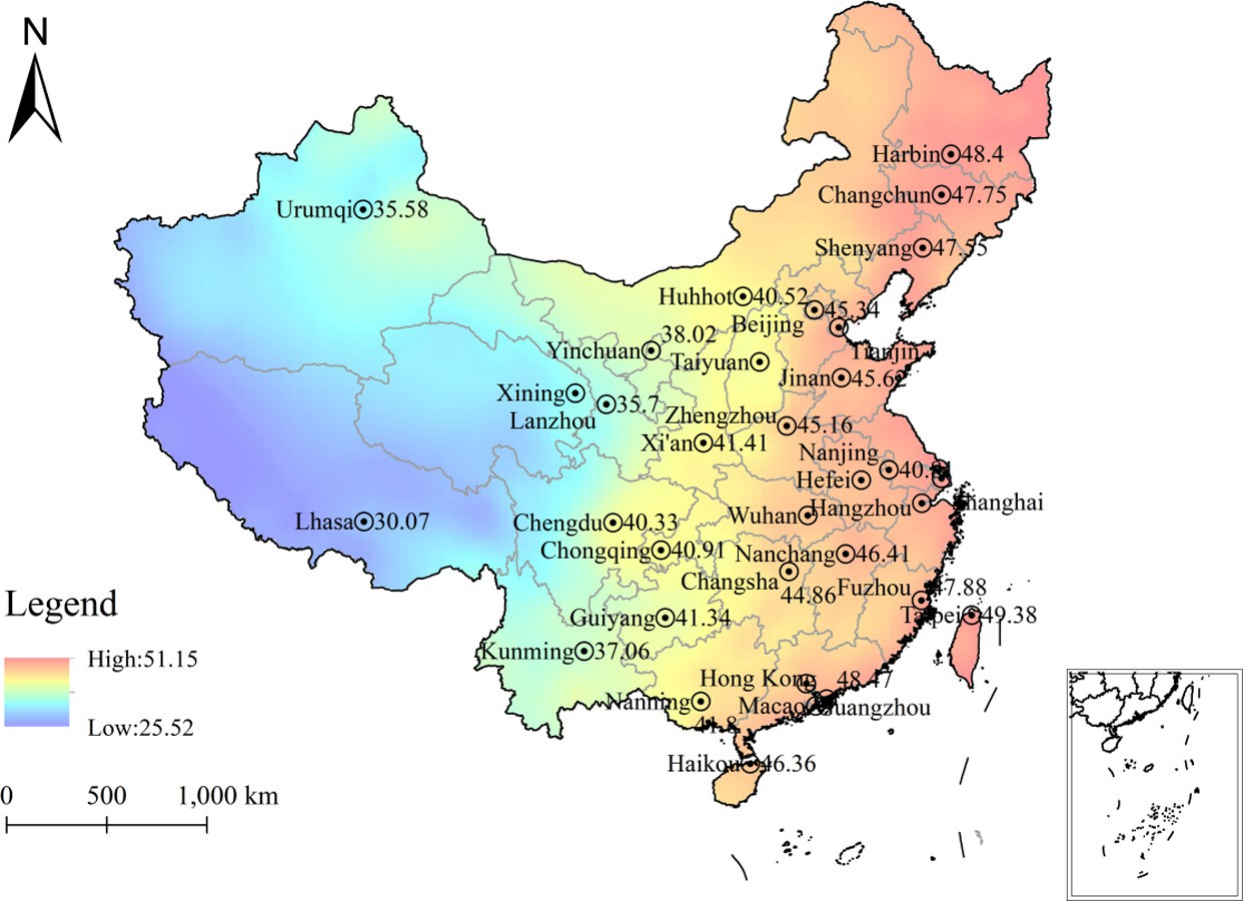
Spatial distribution plot of the predicted RBP values. (This map is based on the standard map with review number GS (2020) 4619, which is publicly available from the website of the Standard Map Service of the Ministry of Natural Resources of China (http://bzdt.ch.mnr.gov.cn/download.html?searchText=GS(2020)4619), and there are no modifications to the base map).

In ArcGIS 10.2, the healthy male RBP reference values of 2322 cities in China obtained by the optimal model SVM prediction were imported and continued in ArcGIS 10.2 for the geostatistical wizard. The RBP reference values for healthy men in 2322 cities were interpolated and mapped, specifically by analytic kriging interpolation (Fig 10). The red part of the figure represents areas with high RBP reference values for healthy men, and the blue part represents areas with low RBP reference values for healthy men. The spatial distribution of RBP reference values for healthy men was found to be gradually increasing from the first to the third order in China. Combined with the distribution map, it is suggested that the RBP reference values for healthy men in China are divided into the first-order low value area (25 mg/L~40mg/L), the second-order middle value area (40mg/L~45mg/L), and the third- order high value area (45mg/L~52mg/L).

## Discussion

Through analytical modeling, according to the generated RBP reference value distribution map of Chinese healthy men, it can be found that there is significant regional variability in the distribution of RBP reference values of Chinese healthy men, and the specific distribution shows a gradually increasing trend from the first to the third order. In combination with geographical factors, three aspects of terrain indicators, climate indicators and soil indicators were discussed.

In terms of terrain indicators, the main influences on RBP reference values for healthy men in China are longitude and altitude, and RBP reference values are positively correlated with longitude and negatively correlated with altitude. That is, from west to east in China, as longitude gradually increases and altitude gradually decreases, the RBP reference value will gradually increase. This is consistent with the findings of Luo et al. [30] which studied the changes in RBP values based on different altitudes and found that RBP values decrease with increasing altitude. This is mainly because the difference in altitude and longitude causes the different climate types in China. Jin [31] studied the RBP values of healthy people in the Nansha region of China, and found that the RBP value decreases with the increase of longitude by comparison.

In terms of meteorological indicators, the two factors that influence the RBP reference values for healthy men in China are the average precipitation and the annual average wind speed, and there is a significant positive correlation between them and these two geographical factors, which indicates that in areas with higher average annual precipitation and average annual wind speed, the RBP reference values for healthy men are correspondingly higher. Jin [31]combined data from health check-ups of healthy military personnel in the Nansha region of China and found that in a high temperature and high humidity environment, as the body temperature and metabolic rate increase, resulting in an increased burden on the kidneys, and in turn, the RBP reference value will be significantly higher than that of the population in a normal environment. This is because the vast areas of the second and third orders of China, which are near the sea and belong to the eastern monsoon region, are significantly influenced by the summer monsoon, have more abundant annual precipitation, high temperature and rainfall, and are flatter than the western part of the terrain, with less ground obstruction, thus creating the characteristic of higher surface wind speed [32]. The first order, i.e., the western region, is inland and higher, forming a highland mountain climate with low temperatures, low precipitation, low humidity and high sunshine hours, and intense ultraviolet radiation making the climate cold and dry [33]. Living in such a low-pressure and low-oxygen environment for a long time, the metabolic rate of the human body will slow down accordingly to maintain itself, which in turn leads to a simultaneous decrease in the filtration function of the kidneys and a decrease in the glomerular filtration rate and renal blood flow, resulting in the storage of RBP in the blood, leading to abnormal RBP concentration in the blood [34, 35]. As the metabolic rate of the human body decreases, it will have a chain effect and thus make the reference value of RBP in the human body at high altitudes smaller [36].

In terms of soil factors, topsoil clay percentage, topsoil reference capacity and topsoil (clay) cation exchange effect RBP reference values in healthy Chinese men, mainly due to the variability of soil development in China. In the first terrace of China, the average altitude is above 4,000 meters, the land types are mostly plateau and desert, and the soil development is slower than that in the east, which in turn causes differences in the percentage of topsoil clay particles, topsoil reference capacity and topsoil (clay) cation exchange under different terraces in China. The different soil types lead to regional differences in the porosity and compactness of soils, making the water, gas and heat exchange between soils affect soil fertility, which in turn affects the growth of crops, so that some natural trace elements in the soil have a corresponding effect on humans through direct radiation or indirectly by entering the food chain [37, 38].

## Conclusion

In this paper, from the perspective of geography, we combined geographic factor data of 2322 cities in China and found that there were relationships between Chinese healthy male RBP reference values and seven geographic factors, namely longitude, altitude, annual precipitation, annual average wind speed, topsoil clay percentage, topsoil reference capacity and topsoil (clay) cation exchange capacity. The RBP reference values of healthy males in 2322 cities in China were predicted by a support vector institution-building prediction model, and the geostatistical analysis revealed that the spatial distribution of RBP reference values of healthy males in China showed a gradually increasing trend from the first order to the third order. Based on the foundation of this study, it is hoped that hydrological factors, individual nutritional intake factors and environmental pollution factors can be introduced in later studies to provide new ideas and a scientific basis for medical development in a more comprehensive manner.

## Supporting information

S1 Data set (XLS)

## References

[1] Blaner WS, Brun Pierre-Jacques, Calderon Rossana M, et al. Retinol-binding protein 2 (RBP2): biology and pathobiology. Crit Rev Biochem Mol Biol, 2020;55:197–218. doi: 10.1080/10409238.2020.1768207 32466661 PMC7593873

[2] Ding QS, Zhang LJ, Zhang QP. Retinol-binding protein-4, insulin resistance, and human metabolic diseases. Chin J Diabetes, 2011;5:429–432.

[3] Zhu YY. Evaluation of the diagnostic significance of urine retinol-binding protein detection in the early injury of renal function. J Clin Lab, 2019;8:160–161.

[4] Yin Q, Zhang YL, Guo SQ, et al. Association between retinol-binding protein 4, hypersensitivity C-reactive protein and diabetic retinopathy. Int J Endocrinol Metab, 2016;36:149–152.

[5] Shen Y. The significance of blood 2 microglobulin in combination with retinol-binding protein detection in early kidney injury. Chin community physicians, 2017;33:117–119.

[6] Wang YQ, Zhang T, Pang WY, et al. The significance of serum adiponectin and retinol binding protein 4 detection in health examination people. Chin Experi Diagnos, 2015;19:291–292.

[7] Zhang JX, Zhu GP, Zhang BL, et al. Elevated serum retinol-binding protein 4 levels are correlated with blood pressure in prehypertensive Chinese. J Human hypertens, 2017;31:611–615. doi: 10.1038/jhh.2017.44 28639612

[8] Wang QY, Tao LN. Progress in retinol-binding protein determination and clinical application. Huaihai Med, 2013;31:3–4.

[9] Xiong JH, Zhao J, Xu S, et al. Diagnostic value of RBP for early renal damage. Med Theory Pract, 2010;23:905–907.

[10] Biesalski HK, Frank J, Beck SC, et al. Biochemical but not clinical vitamin A deficiency results from mutations in the gene for retionol binding protein. Am J Clin Nutr, 1999;69:931–936. 10232633 10.1093/ajcn/69.5.931

[11] Igha tipu Guzili, Muyesenigati. Correlation between retinol-binding protein 4 and lipoprotein A levels and the degree of coronary lesions in elderly Uyghur patients with acute coronary syndrome. China Med Guide, 2017;14:45–49.

[12] Nobuki M, Hirofumi K, Mitsuaki K. Evaluation of the reference range of retinol-binding protein (RBP) levels by the latex turbidimetric immunoassay. Rinsho byori. Jpn J Clin Pathol., 2009;57:195–199. 19363988

[13] Loredana Q, S W B, Leora H, et al. The role of extrahepatic retinol binding protein in the mobilization of retinoid stores. J Lipid Res, 2004;45:1975–1982. 10.1194/jlr.M400137-JLR200 15314099

[14] LS Dall’, PP A, et al. Retinol binding protein in serum and in urine of glomerular and tubular nephropathies. Clin Chim Acta, 1976;68:107–113. doi: 10.1016/0009-8981(76)90409-5 944115

[15] Aktuna D, Buchinger W, Langsteger W, et al. Beta-carotene, vitamin A and carrier proteins in thyroid diseases. Acta Med Austriaca, 1993;20:17–20. 8475673

[16] Wang Z, Yu C, Zhou L, et al. Effects of Tripterygium wilfordii Induction Therapy to IgA Nephropathy Patients with Heavy Proteinuri. Biol Pharm Bull, 2017;40:1833–1838. 28867717 10.1248/bpb.b17-00134

[17] ShenBo C, Yue W, Kokouvi K, et al. Whole-genome sequencing of a Plasmodium vivax clinical isolate exhibits geographical characteristics and high genetic variation in China-Myanmar border area. BMC genomics, 2017;18:131. doi: 10.1186/s12864-017-3523-y 28166727 PMC5294834

[18] Niels D, Bærent AF, Jul MJ, et al. Determinants of vitamin a deficiency in children between 6 months and 2 years of age in Guinea-Bissau. BMC public health, 2013;13:172. doi: 10.1186/1471-2458-13-172 23442248 PMC3599523

[19] A SW, V LR, R TC, et al. Biological monitoring of uranium exposure in south central Virginia. J Exposure Sci Environ Epidemiol, 2008;18:59–75. 10.1038/sj.jes.7500616 17928817

[20] Chen YG. Development of spatial autocorrelation theory and method improvement based on the Moran statistic. Geogr Res Inves, 2009;28:1449–1463.

[21] Su WH. Research on the theory and method of multi-index comprehensive evaluation. Xiamen: Xiamen University, 2000.

[22] Zhang L, Ge M, Wang J. The Influence of geographical environment on the distribution of reference value of tumor marker CYFRA21-1 in Chinese healthy adults. Environ Sci Pollut Res, 2022;29:53168–53175. doi: 10.1007/s11356-022-19617-w 35278178

[23] Marill KA. Advanced statistics: linear regression, part I: simple linear regression. Acad Emerg Med: Off J Soci Acad Emerg Med, 2004;11:87–93. 14709436

[24] Feng GH. SVM classification kernel functions and parameter selection comparison. Comp Eng and Appl, 2011;47:123–124.

[25] Lin SL, Liu Z. SVM parameter selection based on the RBF kernel function. Study Zhejiang Univ Technol Newspaper, 2007;12:163–167.

[26] Wang Q, Leng LF, Chang YL. Stock index tracking study improved ridge regression and principal component regression. Chongqing J University Sci Technol, 2018;32:212–321.

[27] Taylor KE. Summarizing multiple aspects of model performance in a single diagram. J Geophys Res: Atmos, 2001;106, 7183–7192.

[28] Li RQ. Application of ensemble dynamics factors in Inner Mongolia. Inner Mongolia Gas Elephant, 2018;5:3–7.

[29] Jalloh AB, Kyuro S, Jalloh Y, et al. Integrating artificial neural networks and geostatistics for optimum 3D geological block modeling in mineral reserve estimation: A case study. Int J Min Sci Technol, 2016;26:581–585.

[30] Luo PL, Ba YG, Du WJ. Effects of acute plateau hypoxia on urinary low molecular protein[J]. J Qinghai Med College, 2007, 03:198–199.

[31] Jin YM, LI B, Chen R, et al. Effects of high-intensity military training on the kidneys under high temperature and high humidity environment[J]. J Naval Med, 2021, 42:403–406.

[32] Wang N, You QL, Liu JJ. Long-term change trend of ground wind speed in China in 1979–2014. J Nat Res, 2019;34:1531–1542.

[33] Liu XD, Yan LB, Cheng ZG, et al. The dependence of mountain climate warming on altitude in the middle and low latitudes. Plateau mt Meteorol Res, 2008;1:19–23.

[34] Li XL, Su T, Xiao H, et al. Association of the HDL-c Level with HsCRP, IL-6, U-NAG, RBP and Cys-C in Type 2 Diabetes Mellitus, Hypertension, and Chronic Kidney Disease: An Epidemiological Survey. Diabetes Metab Syndr Obes: Targets Ther, 2020;13:3645–3654. doi: 10.2147/DMSO.S265735 33116716 PMC7568590

[35] Lian L, Gu XL. Application of multiple serum combinations in diabetic nephropathy. Laboratory Med and Clini, 2011;8:2565–2566.

[36] Wu J, Shao XH, Lu K, et al. Urinary RBP and NGAL Levels are Associated with Nephropathy in Patients with Type 2 Diabetes. Cellular Physiol Biochemi, 2017;42:594–602. doi: 10.1159/000477860 28954270

[37] Wen YC, Li YQ, Yuan L, et al. Comprehensive evaluation method of soil fertility characteristics of different long-term fertilization systems agriculture. J Ind Eng, 2015;31:91–99.

[38] Jiang JQ. The Relationship between Environmental Geochemistry and Human Health and Agricultural Production. Geophys chem explor, 2004;28:330–332.

